# Physical activity levels and determinants of change in young adults: a longitudinal panel study

**DOI:** 10.1186/1479-5868-7-2

**Published:** 2010-01-11

**Authors:** Dorith Zimmermann-Sloutskis, Miriam Wanner, Erwin Zimmermann, Brian W Martin

**Affiliations:** 1 Swiss Federal Institute of Sport Magglingen, Hauptstrasse, 2532 Magglingen, Switzerland; 2 Formerly Swiss Household Panel, Department of Humanities and Social Sciences, University of Neuchâtel, Espace de l'Europe 4, 2002 Neuchâtel, Switzerland; 3 Institute of Social and Preventive Medicine, University of Zurich, Hirschengraben 84, 8001 Zurich, Switzerland

## Abstract

**Background:**

There is growing concern about physical inactivity in adolescents and young adults. Identifying determinants that are associated with low levels of physical activity and with changes in physical activity levels will help to develop specific prevention strategies. The present study describes the prevalence and potential determinants of physical activity behavior and behavior changes of young adults. The study is based on the Swiss Household Panel (SHP), a longitudinal study assessing social changes in a representative sample of Swiss households since 1999.

**Methods:**

Data is collected yearly using computer-assisted telephone interviews. Information is obtained from each household member over 14 years of age. Participants between 14 and 24 years entering the SHP between 1999 and 2006 were included (N = 3,068). "Inactive" was defined as less than 1 day/week of at least 30 minutes of moderate physical activity, "no sport" as exercising less than once a week. Age, gender, nationality, linguistic region, household income, education, membership in a sport club, reading, and Internet use were included as potential determinants of physical activity behavior and behavior change.

**Results:**

In both young men and young women, the prevalence of inactivity, "no sport", and non-membership in a sport club was increasing with age. Women were less active than men of the same age. From one wave to the following, 11.1% of young men and 12.1% of young women became active, and 11.9% of men and 13.7% of women became inactive, respectively (pooled data over all eight waves). Non-membership in a sport club was the strongest predictor for "no sport" (OR_men _6.7 [4.9-8.9]; OR_women _8.1 [5.7-11.4]), but also for being inactive (OR 4.6 [3.5-6.0]; 4.6 [3.3-6.4]). Leaving a sport club (OR 7.8 [4.4-14.0]; 11.9 [5.9-24.1]) and remaining non-member (OR 7.8 [4.7-12.9]; 12.4 [6.4-24.1]) were the strongest predictors of becoming "no sport". Effects for becoming inactive were similar, though smaller (OR 5.9 [3.4-10.5] and 5.1 [2.7-9.6] for leaving a club, OR 5.1 [3.1-8.4] and 6.9 [4.0-11.8] for remaining non-member).

**Conclusions:**

The most important findings were the strong effects of sport club membership on general physical activity. The correlation between sport club membership and exercise was not surprising in its nature, but in its strength.

## Background

The importance of physical activity for health has been well documented both at the individual and at the population level [1]; it has also been accepted by international public health authorities [2,3]. Current recommendations point out the importance of regular, ideally daily, physical activity of at least moderate intensity and the additional benefits of activities of vigorous intensity [4,5]. While half an hour of moderate intensity physical activity is recommended for adults, adolescents should accumulate at least one hour per day [6].

At the same time, there is growing concern about low levels of physical activity in many countries [7,8]. There are only a limited number of countries that can follow physical activity trends over several years based on national representative data. Most of these time series are based on repeated independent cross-sectional studies [9,10] potentially confounded by cohort effects. While there is a number of longitudinal studies on physical activity behavior [11], only few of them are based on representative population samples of sub-national regions [12] or even countries [13,14].

In Switzerland, nationally representative data on physical activity in adults is available since the 1992 Swiss Health Survey [15], data relating to current recommendations for health-enhancing physical activity for the 2002 and 2007 Swiss Health Survey [6,16]. With a prevalence of 41% sufficiently active individuals in 2007 they are in line with international estimates of physical inactivity. Young people report more physical activity than older ones, men more than women. The proportion of individuals with less than one vigorous exercise episode per week could be followed over 15 years: it increased from 36% to 39% between 1992 and 1997, but then decreased again to 37% by 2002 and to 32% by 2007 [6]. More detailed information was available for 1997, 2002 and 2007: participation in sports and exercise remained stable between 1997 and 2002 (and even increased in women), and increased slightly between 2002 and 2007. In both sexes the proportion walking or using their bicycle for daily transport decreased from 57.5% in 1997 to 49.5% in 2002 but increased again to 56.4% by 2007 [6,17].

Data from cross-sectional studies indicate that the largest declines in physical activity levels with age occur in adolescents and young adults and above the age of 65 years [16]. Decreasing physical activity levels at younger ages are of special concern. Several determinants can influence physical activity behavior [2]. These can be non-modifiable, such as age, gender, and social class, or modifiable. Modifiable determinants include personal factors (e.g. attitudes, motivation, self-efficacy), the social environment (e.g. family, peers, social support), and the physical environment (e.g. access to sport facilities, urban development, transport infrastructure, green spaces). Identifying determinants that are associated with changes in physical activity will allow to develop specific and effective prevention strategies for specific groups, for example adolescents and young adults. In a review of longitudinal studies on youth predictors of adulthood physical activity [11], associations were described with a number of variables in youth. The strongest associations were reported for high levels of physical activity, frequent participation in sport, membership in a sport club, good cardio-respiratory fitness and high marks in physical education at school. In a recent review of quantitative reviews for the National Centre on Clinical Excellence NICE [18], the following demographic, behavioral and cultural correlates of physical activity in children and adolescents have been identified: male gender (moderate to large positive association), age in adolescence (small to moderate negative), previous physical activity (moderate), sport participation (moderate, stronger in adolescent girls), smoking (moderate negative), and parental and social support (large positive).

Recent epidemiologic evidence suggests that too much sitting may be a cardiovascular and metabolic risk factor that is independent of physical inactivity [19]. Moreover, sedentary behavior has been a concern in children and adolescents. According to displacement hypotheses, spending time in sedentary activities reduces the time spent participating in physical activity. Sedentary behavior after school and on weekends was inversely related to physical activity in adolescents [20]. However, the relation between sedentary behavior in general (mostly studied based on television viewing or as a collapsed variable based on different sedentary behaviors) and physical activity was indeterminate in children and adolescents [20]. This may be due to the fact that different types of sedentary behaviors show different associations with physical activity behavior. While watching TV has been reported to inversely correlate with physical activity in a meta analysis [21], the literature regarding computer and internet use is equivocal: Inverse associations with physical activity have been reported for computer use/playing video games [13] and for the availability of computers/Internet at home [22], positive associations for working on computers [23] and computer use [24,25], especially in young men [26], and no associations for leisure time internet use in adolescent girls [27,28]. While reading/doing homework was not associated with physical activity, a combination of "productive" sedentary behaviors including reading/doing homework and working on a computer showed a positive correlation with physical activity behavior [23]. Two other studies reported a significant positive association between reading and physical activity in both young men and young women [25] and in young women only [26].

In Switzerland, sport clubs are a central part of the sport structure and play an important role in sport promotion, especially in younger age groups. The proportion of sport club members is 62% in children aged 10-14 years and 47% in adolescents aged 15-19 years [29]. Sport clubs are organized locally in towns and villages, offering trainings for different age groups at least once a week. Usually supervised by qualified volunteer instructors, sport clubs are open to everyone at very low annual fees. Leaving a sport club at the crucial age of adolescence may be a factor, among others, associated with decreasing activity levels between youth and adulthood in Switzerland and other countries that have a tradition of sport clubs.

In repeated cross-sectional studies changes are only followed on the population level, so behavioral changes in individuals and groups that cancel out each other might go unnoticed. Longitudinal studies, such as the Swiss Household Panel (SHP), give the opportunity to describe behavior and changes in behavior at the individual level. They allow to gain estimates of the proportion of spontaneous behavioral changes to be expected in subgroups of the population and to assess determinants of physical activity behavior and its changes. Longitudinal panel studies investigate several birth cohorts on repeated occasions. This publication describes the prevalence of physical activity of moderate and vigorous intensity among adolescents and young adults aged 14 to 24 years when entering the panel over up to 8 years of follow-up from 1999 to 2006. As well, it will quantify the role of potential determinants of physical activity behavior and of changes in physical activity behavior.

## Methods

### Study population

The Swiss Household Panel is a longitudinal nationwide survey assessing living conditions and social changes in a representative sample of Swiss households since 1999. The panel is described in detail elsewhere [30]. Briefly, data is collected every year using centralized computer-assisted telephone interview (CATI) techniques. General information on the socio-demographic situation of the household and the household composition is provided by an adult reference person in each household. Individual information is obtained from each household member over 14 years of age. Questions include socio-demographic variables, health, well-being, political attitudes and behavior, social networks and economic issues. The Swiss Household Panel was approved by the Research Commission of the University of Neuchâtel, which grants the conformity to the ethical standards of research on humans. Every year, each selected household receives a letter containing information about the panel, its aims, the length and content of the interviews, as well as the confidentiality, anonymity and exclusive use of the data for scientific research purposes. A few days later the survey institute contacts the households by telephone, and each member is free to participate or refuse participation.

The first wave of the panel started in 1999, when 14,174 households were randomly selected from the electronic telephone registry. Of these, 7,063 households could be reached for basic information and 6,001 answered the household questionnaire. In 5,074 households with a total of 10,293 eligible individuals above 14 years of age, at least one individual interview was completed resulting in 7,799 individual interviews (75.8%). Of the 7,799 individuals, 1,159 were young participants aged 14-24 years living in 842 households. Children 13 years and less in 1999 entered the panel as soon as they turned 14 years. Occasionally, some older subjects entered the panel at a later time point as well, for example because they returned to their household. In 2004, a new sample was added to the panel to compensate for attrition (more information on the Swiss household panel and the selection methods are available at http://www.swisspanel.ch).

The inclusion criteria for the present study were a) to be born between 1975 and 1992, b) to be between 14 and 24 years of age at the year of inclusion and c) to be between 14 and 26 years of age at the time of the interview for the respective wave. At the start of the panel in 1999, 1,159 eligible adolescents and young adults participated in the individual interviews. Due to the refreshment of the panel in 2004, 740 individuals entered the panel in that year. Between 125 and 305 individuals entered the panel in the remaining years (2000, 2001, 2002, 2003, 2005, and 2006, see Table 1), resulting in a total sample of 3,068 individuals (50.0% males, N = 1,534). These relatively high numbers of new entries of young individuals every year are due to the design of the panel that includes individuals from the age of 14 years on. Thus any child turning 14 would enter the panel in the respective year. Repeated participation of the 3,068 individuals resulted in 9,039 observations. The proportion of individuals providing data for 1 to 8 waves, respectively, were 29.1%, 19.8%, 18.9%, 7.8%, 8.2%, 5.9%, 5.0%, and 5.4%. The study adherence in the sample of adolescents and young adults aged between 14 and 26 years included in the present study is summarized in Table 1. We are aware that by following individuals over time using a panel design, the initial age at entry is not fully determined by the birth cohort because not all participants started in 1999.

**Table 1:** Sample size, adherence, loss and (re-) recruitment of participants at each wave

		Adherence and loss of participants		recruitment and re-recruitment	
		
	total N	individuals who participated in previous wave	individuals lost since previous wave	New Individuals entering the panel	Individuals who participated before but not in previous wave
wave 1 - 1999	1159			1159	
wave 2 - 2000	1148	843	316 (27.3%)	305	
wave 3 - 2001	1134	865	283 (24.7%)	194	75
wave 4 - 2002	995	786	348 (30.7%)	125	84
wave 5 - 2003	915	721	274 (27.5%)	133	61
wave 6 - 2004	1373	564	351 (38.4%)	740	69
wave 7 - 2005	1149	879	494 (36.0%)	205	65
wave 8 - 2006	1166	807	342 (29.8%)	207	152

Note: In total, 1534 males and 1534 females were included in the analyses.

### Measures

#### Physical activity

Self-reported physical activity was assessed using two questions on activities with moderate intensity (number of days of at least 30 minutes) and one question on frequency of sport or exercise practiced individually or in a team. "Activities that get you at least a little bit out of breath" such as walking quickly, dancing, or gardening were named as examples of moderate activities; jogging, football, volleyball, and tennis as examples of sport activities and exercise. No minimum duration of exercise participation was taken into account. It was not possible to include a more detailed questionnaire on physical activity behavior because this was only a minor topic in the Swiss Household Panel. Three dichotomous physical activity outcome measures were defined for activities of moderate and vigorous intensity and a combination of both: "inactive" was defined as less than one day of at least 30 minutes of moderate physical activity per week, "no sport" as exercising less than once a week. Finally the combination of "inactive" and "no sport" was defined as "completely inactive". The distribution of individuals in the different levels of moderate and vigorous intensity activities is shown in Table 2.

**Table 2:** Levels of moderate and vigorous physical activity at initial age when entering the study

		Males (N = 1534)	Females (N = 1534)
number of days with ≥ 30 min of moderate activities per week			

**inactive**	**0 day**	**23.4%**	**29.7%**
	1 day	22.6%	25.9%
	2 days	11.5%	12.0%
	3 days	27.7%	21.1%
active	4 days	6.5%	3.9%
	5 days	3.8%	3.6%
	6 days	1.7%	1.5%
	7 days	2.8%	2.3%

frequency of engaging in sport activities			

**"no sport"**	**never**	**9.6%**	**17.9%**
	**less than once a month**	**2.6%**	**3.2%**
	**at least once a month**	**6.6%**	**8.9%**
active in sport	at least once a week	69.4%	63.2%
	every day	11.8%	6.8%

frequency of complete inactivity			

**completely inactive**	**inactive and no sport**	**12.6%**	**20.6%**
somewhat active		87.4%	79.4%

The categories printed in bold correspond to the definition of the dichotomous physical activity outcome variables.

#### Potential determinants of physical activity

Time-invariant and time-variant determinants were included in the analysis based on indications of existing cross-sectional studies. Time-invariant determinants included gender, initial age when entering the study, nationality (Swiss nationality versus no Swiss nationality), linguistic region (German, French or Italian speaking part) and size-adjusted household income over all waves (quintiles, adjusted for number and age of household members) as a proxy for socio-economic status. The lowest quintile of the size-adjusted household income represents an annual income of ≤ 33,070 Swiss Francs (around 22,000 Euro). Time-variant determinants at any time point included individual current age at each wave, membership in a sport club, education, Internet use, and reading. Education was included as reported at the time of the survey or as the highest education achieved if education was completed (compulsory school up to age 16; high school or apprenticeship/vocational low, typically from 16 to 20 years; vocational high/university from 21 to 26 years). Internet use as a recently emerged behavior and reading as a more traditional one were included as leisure time activities representing sedentary behavior (frequency of engaging in activity during leisure time, more than once a week versus less). TV viewing was only measured in 1999 and was not included in the analyses. Due to its strong correlation with age, education was only included in the univariate analyses but not in the adjusted models.

### Statistical analyses

For modeling the likelihood of binary outcomes as for low level of physical activity ("inactive", "no sport", "completely inactive"), a generalized estimating equation (GEE) model with a logistic link function and a binomial distribution was applied. Determinants of low levels of physical activity were estimated using pair-wise log odds ratios for the within-subject association. Determinants of change were analyzed applying an independent correlation structure for the outcome conditional to previous physical activity level (change in physical activity). The GEE method is a generalization of the general linear model (GLM) that permits to take into account within-subjects dependencies. The PROC GENMOD command in SAS was used. Estimates are reported as crude and adjusted odds ratios (OR) and their 95% confidence intervals (95% CI) for males and females separately.

The data were weighted in order to represent the population living in private households in Switzerland. Because of the panel study design, cross-sectional as well as longitudinal weights had to be applied. Cross-sectional weights extrapolated data to the population composition in each survey year and were re-scaled to keep the original sample size of the young population. Longitudinal weights extrapolated to the population resident in Switzerland in 1999 and in 2004 (refreshment sample introduced in 2004), respectively. The variables used in creating weights were gender, age, Swiss region and nationality (more details on the technical descriptions of the weights can be found at http://www.swisspanel.ch/doc/methodology.php?lang=en&pid=8). All analyses were carried out using SAS Version 9.1.

## Results

### Prevalence of physical activity behavior and of potential determinants

Table 3 shows the prevalence of physical inactivity, sport club membership and education by age category. In both young men and women, the prevalence of inactivity, "no sport", complete inactivity, and non-membership in a sport club was increasing with age, particularly in the three younger age groups. Comparing genders, women were clearly less active than men of the same age group, particularly regarding exercising and sport club membership. Over all age groups, the median size-adjusted household income was 47,000 Swiss Francs (29,000 Euro). In the sample, 83.0% had Swiss nationality, including 11.7% with a second nationality. 26.8% were living in the French speaking, 69.7% in the German speaking and 3.5% in the Italian speaking region of Switzerland. In terms of sedentary activities, 62.0% of participants were using the Internet every day and 49.9% were reading every day.

**Table 3:** Prevalence of physical inactivity, sport club membership and education by gender and age categories

	Males					Females				
**Age**	**14-16**	**17-19**	**20-22**	**23-26**	**all ages^1^**	**14-16**	**17-19**	**20-22^2^**	**23-26**	**all ages^1^**

**N of observations**	**1322**	**1257**	**1026**	**909**	**4514**	**1332**	**1250**	**986**	**957**	**4525**

**Physical inactivity**										
% inactive^2^	19.0	22.9	31.7	26.7	24.8	24.6	29.2	34.5	36.3	30.6
% "no sport"^2^	13.6	22.0	28.1	28.1	22.6	23.0	30.1	37.6	37.1	31.2
% completely inactive^2^	8.3	13.8	21.6	20.0	15.6	14.4	20.9	26.7	29.2	22.1

**Sport club membership**										
% members^2^	63.2	56.2	49.3	47.1	54.3	49.6	41.0	30.2	32.1	39.2

**Education**										
compulsory school	67.0	0.0	0.0	0.0	17.3	65.9	0.0	0.0	0.0	18.7
high school	12.6	20.8	2.7	0.4	9.6	15.9	29.5	5.2	0.3	13.9
apprenticeship, vocational low	20.4	74.0	60.1	47.5	50.7	18.2	63.8	56.3	51.1	46.5
vocational high	0.0	1.1	11.1	19.4	7.4	0.0	1.1	7.0	12.0	4.4
university	0.0	4.1	26.2	32.7	15.0	0.0	5.6	31.6	36.6	16.5

Pooled data over all eight waves (1999-2006)

^1 ^all p-values from Wald test (GEE) comparing age categories were significant in males and in females (p < 0.0001)

^2 ^all p-values from Wald test (GEE) comparing men and women within age categories were significant for no sport (p < 0.005), for completely inactive (p < 0.04), and for membership in a sport club (p < 0.0001). For inactive, p-values were significant (p < 0.005) except in the age group 20-22 years (p = 0.3).

### Prevalence of changes in physical activity behavior

Analysis of the pooled data over all eight waves resulted in 11.1% of young men and 12.1% of young women becoming active from one wave to the following, and in 11.9% of men and 13.7% of women becoming inactive, respectively. 8.9% of men and 12.4% of women became active in sport, while 11.7% and 14.1% changed their exercise status to "no sport", respectively. In men, 7.1% became somewhat active and 9.4% became completely inactive. The corresponding numbers for women were 9.8% and 11.1%, respectively.

**Figure 1:**
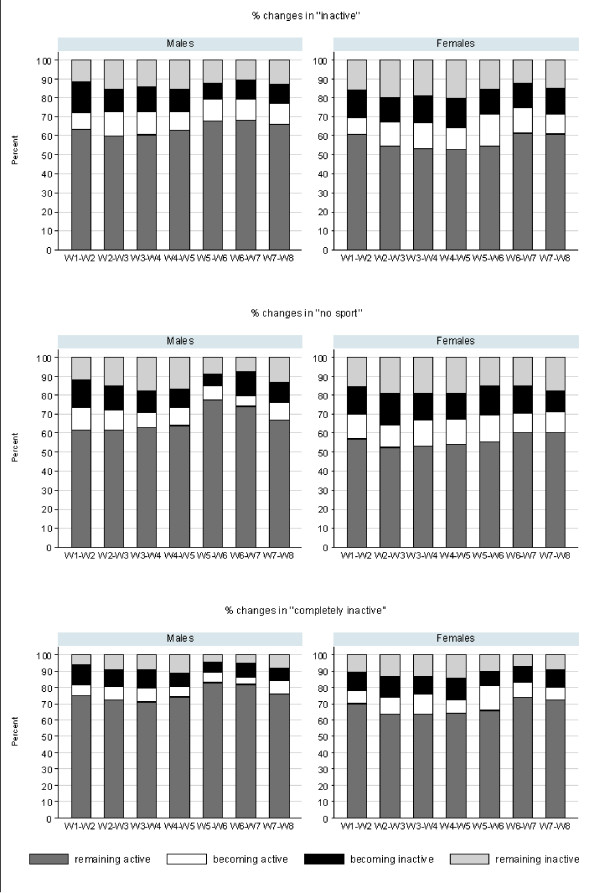
**Prevalence of changes in physical activity behavior by gender and wave, SHP 1999-2006**. Legend: W1 = 1999, W2 = 2000, W3 = 2001, W4 = 2002, W5 = 2003, W6 = 2004, W7 = 2005, W8 = 2006. There were significant differences between waves in the distribution of individuals within the four categories of changes in "no sport" in men (p < 0.001) and of changes in "inactive" in women (p = 0.03). In both genders the distribution of changes in "completely inactive" was significantly different between waves (p < 0.01).

Fig. 1 shows the proportions of changes in inactive, "no sport", and completely inactive between any two consecutive non-missing waves for young men and young women. For all waves, the proportion of physically active individuals who remained active (remaining active, remaining active in sport, remaining somewhat active) were higher in men than in women. The proportion of physically inactive individuals who remained inactive (remaining inactive, remaining "no sport", remaining completely inactive) were higher in women than in men. The overall spontaneous changes from active to inactive and vice versa were relatively stable over waves and similar in men and women, ranging mostly from around 10% to 15%.

### Potential determinants for physical activity behavior and behavior change

To determine risk factors for inactivity and "no sport" and for changes in physical activity behavior, selected time-variant and time-invariant determinants were included in the analysis. Table 4 shows the OR with 95% CI and the respective p-values for inactivity and "no sport" for each variable separately (unadjusted) and adjusted simultaneously for all variables except education.

**Table 4:** Odds ratios for being physically inactive in young males and females

	Men				Women			
	
	"no sport"		inactive		"no sport"		inactive	
	
	unadjusted	adjusted^1^	unadjusted	adjusted^1^	unadjusted	adjusted^1^	unadjusted	adjusted^1^
Age category								
14-16 years	1.0	1.0	1.0	1.0	1.0	1.0	1.0	1.0
17-19 years	1.7 (1.3-2.2)	1.7 (1.2-2.3)	1.2 (0.9-1.5)	1.2 (0.8-1.6)	1.5 (1.3-1.8)	1.5 (1.1-2.1)	1.3 (1.1-1.5)	1.6 (1.1-2.2)
p-value	<0.001	0.007	0.07	0.3	<0.001	0.03	0.004	0.02
20-22 years	2.3 (1.8-2.9)	1.9 (1.4-2.8)	1.8 (1.5-2.3)	1.8 (1.2-2.4)	1.9 (1.5-2.4)	1.8 (1.3-2.6)	1.5 (1.2-1.9)	1.8 (1.2-2.5)
p-value	<0.001	0.002	<0.001	0.003	<0.001	0.001	<0.001	0.006
23-26 years	2.2 (1.7-2.9)	2.3 (1.5-3.5)	1.4 (1.1-1.8)	1.3 (0.9-2.0)	1.8 (1.4-2.3)	1.5 (1.1-2.2)	1.7 (1.3-2.1)	1.7 (1.2-2.6)
p-value	<0.001	<0.001	0.02	0.2	<0.001	0.04	<0.001	0.009

Sport club membership								
member	1.0	1.0	1.0	1.0	1.0	1.0	1.0	1.0
non-member	6.6 (5.4-8.1)	6.7 (4.9-8.9)	4.2 (3.5-5.0)	4.6 (3.5-6.0)	7.3 (6.0-8.9)	8.1 (5.7-11.4)	5.3 (4.4-6.5)	4.6 (3.3-6.4)
p-value	<0.001	<0.001	<0.001	<0.001	<0.001	<0.001	<0.001	<0.001

Education								
compulsory school	1.0		1.0		1.0		1.0	
high school	1.1 (0.8-1.5)		0.7 (0.5-1.0)		1.2 (0.9-1.6)		1.0 (0.8-1.3)	
p-value	0.8		0.04		0.2		0.9	
apprenticeship, vocational low	2.1 (1.6-2.7)		1.5 (1.2-1.9)		2.2 (1.8-2.7)		1.9 (1.6-2.3)	
p-value	<0.001		<0.001		<0.001		<0.001	
vocational high	2.0 (1.5-2.8)		1.6 (1.2-2.3)		1.2 (0.8-1.7)		1.0 (0.7-1.5)	
p-value	<0.001		0.003		0.4		0.8	
University	2.1 (1.6-3.0)		1.5 (1.1-2.0)		1.3 (1.0-1.8)		1.3 (0.9-1.6)	
p-value	<0.001		0.01		0.04		0.08	

Household Income								
Four higher quintiles	1.0	1.0	1.0	1.0	1.0	1.0	1.0	1.0
Lowest quintile	1.3 (0.9-1.7)	1.3 (0.9-1.9)	1.6 (1.2-2.0)	1.4 (1.0-2.1)	1.4 (1.1-1.8)	1.0 (0.7-1.4)	1.5 (1.2-2.0)	1.0 (0.7-1.4)
p-value	0.1	0.1	<0.001	0.03	0.007	0.5	0.002	0.9

Nationality								
Swiss	1.0	1.0	1.0	1.0	1.0	1.0	1.0	1.0
Non-Swiss	1.2 (0.8-1.6)	0.9 (0.5-1.5)	1.5 (1.1-2.0)	1.2 (0.8-2.0)	2.6 (1.9-3.4)	1.7 (1.1-2.7)	2.8 (2.1-3.6)	2.1 (1.3-3.2)
p-value	0.3	0.6	0.007	0.4	<0.001	0.02	<0.001	0.0009

Language region								
German-speaking	1.0	1.0	1.0	1.0	1.0	1.0	1.0	1.0
French-speaking	1.1 (0.9-1.4)	1.1 (0.8-1.6)	1.7 (1.4-2.2)	1.6 (1.2-2.3)	1.5 (1.2-1.9)	1.4 (1.0-1.9)	2.4 (2.0-3.0)	2.2 (1.6-2.9)
p-value	0.3	0.6	<0.001	0.004	<0.001	0.04	<0.001	<0.001
Italian-speaking	1.9 (1.1-3.5)	1.7 (0.7-3.8)	3.5 (2.1-5.8)	3.2 (1.6-6.2)	2.6 (1.5-4.3)	1.8 (0.9-3.7)	3.1 (2.0-4.8)	2.1 (1.1-3.8)
p-value	0.03	0.2	<0.001	<0.001	<0.001	0.2	<0.001	0.03

Internet use								
Rarely-never	1.0	1.0	1.0	1.0	1.0	1.0	1.0	1.0
Every day	0.7 (0.6-0.9)	0.7 (0.5-0.9)	0.8 (0.7-1.1)	0.8 (0.6-1.0)	0.6 (0.5-0.8)	0.6 (0.5-0.8)	0.8 (0.7-1.1)	0.8 (0.6-1.1)
p-value	0.04	0.02	0.08	0.07	<0.001	<0.001	0.1	0.1

Reading								
Rarely-never	1.0	1.0	1.0	1.0	1.0	1.0	1.0	1.0
Every day	1.0 (0.8-1.1)	1.0 (0.8-1.3)	1.0 (0.9-1.2)	1.0 (0.8-1.3)	0.9 (0.8-1.0)	0.7 (0.6-0.9)	0.9 (0.8-1.0)	0.8 (0.6-0.9)
p-value	0.7	0.9	0.9	0.9	0.06	0.02	0.05	0.02

^1 ^adjusted for all variables displayed in the table except education

All estimates and 95% CI are based on the pooled data using the GEE model with pair-wise log odds ratios for the within-subject correlation, n_male participants _= 1,534; n_female participants _= 1,534)

Age higher than 16 years was a significant predictor for "no sport" in both genders and for "inactive" in women. In both genders, the (unadjusted) risk-enhancing effect of not being a member in a sport club was considerable for "inactive" (OR_men _4.2, 95% CI 3.5-5.0; OR_women _5.3, 95% CI 4.4-6.5) and even more so for "no sport" (OR_men _6.6, 95% CI 5.4-8.1; OR_women _7.3, 95% CI 6.0-8.9). In the full model adjusting simultaneously for all potential determinants (except education), the effect of sport club membership became even stronger (see Table 4). There was a tendency towards higher risks of inactivity and "no sport" among individuals living in households in the lowest quintile of income, however most OR were not significant. In the adjusted model, Non-Swiss nationality was a significant predictor for "inactive" (OR 2.1, 95% CI 1.3-3.2) and "no sport" (OR 1.7, 95% CI 1.1-2.7) in females only. The risk of being "inactive" was significantly higher in the French and Italian speaking regions compared to the German speaking one in men and in women even after adjusting for covariates. For "no sport" in the adjusted model, the risk was higher only for females in the French speaking part (borderline significance). Daily internet use was a protective factor for both outcomes and both genders (though borderline significant for "inactive" in males and non-significant for "inactive" in females) when adjusted for covariates. Daily reading was protective only in women. If education was also introduced in the multivariate model, all age categories became non-significant for both outcomes.

Table 5 shows the risk of becoming "no sport" or "inactive" for previously active individuals, including selected determinants, unadjusted and adjusted for all variables except education. In the adjusted model, age was no longer a significant predictor for becoming "inactive" or "no sport", neither in men nor in women. The risk of becoming "inactive" or "no sport" was clearly higher (with OR of up to 12) in both individuals who remained outside a sport club and those who had left one in the year preceding the observation (see Table 5). Compared to individuals who remained outside a sport club, those who had joined one had a slightly elevated risk of becoming inactive (men only, OR 2.7, 95% CI 1.1-6.3) and "no sport" (women only, OR 2.7, 95% CI 1.1-7.0), respectively. A lower household income was a borderline significant predictor for changing the exercise status to "no sport" in young men (OR 1.9, 95% CI 1.0-3.5). Nationality was not a significant predictor in the adjusted model neither in young men nor in young women. For young women, the risk of becoming "inactive" and "no sport" was significantly higher in the Italian speaking compared to the German speaking part of Switzerland. In the French speaking part the risk was only elevated for becoming "inactive". For young men, neither risk was related to linguistic region in the adjusted model. Daily internet use was a protective factor for becoming "inactive" and "no sport" in women but not in men. Daily reading was not associated with changes neither for "no sport" nor for "inactive" in both genders.

**Table 5:** Odds ratios for becoming physically inactive in previously active young males and females

	Men				Women			
	
	becoming "no sport"		becoming inactive		becoming "no sport"		becoming inactive	
	
	unadjusted	adjusted^1^	unadjusted	adjusted^1^	unadjusted	adjusted^1^	unadjusted	adjusted^1^
Age category								
14-16 years	1.0	1.0	1.0	1.0	1.0	1.0	1.0	1.0
17-19 years	1.7 (1.1-2.4)	1.7 (0.9-3.3)	1.3 (0.9-1.9)	1.0 (0.5-1.8)	0.9 (0.7-1.3)	0.9 (0.5-1.6)	1.1 (0.8-1.5)	1.2 (0.8-2.0)
p-value	0.01	0.1	0.2	0.9	0.7	0.9	0.8	0.3
20-22 years	2.0 (1.3-3.0)	1.7 (0.9-3.3)	1.8 (1.1-2.6)	1.7 (0.9-3.1)	1.1 (0.8-1.5)	1.1 (0.6-2.1)	1.3 (0.9-1.8)	1.4 (0.8-2.6)
p-value	<0.001	0.1	0.003	0.07	0.5	0.6	0.2	0.2
23-26 years	1.9 (1.2-2.9)	1.8 (0.9-3.5)	1.3 (0.9-2.0)	1.2 (0.6-2.4)	1.1 (0.7-1.5)	0.7 (0.4-1.3)	1.4 (1.0-2.0)	1.5 (0.8-2.6)
p-value	0.004	0.1	0.2	0.6	0.7	0.3	0.05	0.2

Sport club membership								
remaining member	1.0	1.0	1.0	1.0	1.0	1.0	1.0	1.0
becoming member	1.4 (0.7-2.7)	1.3 (0.4-3.3)	2.1 (1.2-3.7)	2.7 (1.1-6.3)	1.5 (0.8-2.8)	2.7 (1.1-7.0)	2.1 (1.2-3.6)	1.6 (0.7-3.7)
p-value	0.3	0.6	0.01	0.02	0.2	0.04	0.007	0.2
becoming non-member	7.4 (4.9-11.0)	7.8 (4.4-14.0)	5.6 (3.9-8.1)	5.9 (3.4-10.5)	7.0 (4.5-11.1)	11.9 (5.9-24.1)	5.4 (3.5-8.5)	5.1 (2.7-9.6)
p-value	<0.001	<0.001	<0.001	<0.001	<0.001	<0.001	<0.001	<0.001
remaining non-member	9.2 (6.6-13.1)	7.8 (4.7-12.9)	5.2 (3.7-7.4)	5.1 (3.1-8.4)	10.7 (7.3-15.6)	12.4 (6.4-24.1)	7.9 (5.4-11.3)	6.9 (4.0-11.8)
p-value	<0.001	<0.001	<0.001	<0.001	<0.001	<0.001	<0.001	<0.001

Education								
compulsory school	1.0		1.0		1.0		1.0	
high school	0.4 (0.2-0.8)		0.4 (0.2-0.8)		0.8 (0.5-1.3)		1.0 (0.6-1.7)	
p-value	0.004		0.004		0.3		0.9	
apprenticeship, vocational low	1.7 (1.1-2.7)		1.4 (0.9-2.3)		1.4 (0.9-2.0)		1.9 (1.3-2.9)	
p-value	0.01		0.07		0.1		0.001	
vocational high	1.4 (0.7-2.6)		1.7 (0.9-3.2)		1.0 (0.5-1.9)		1.2 (0.6-2.4)	
p-value	0.5		0.06		0.7		0.9	
University	1.4 (0.8-2.3)		1.0 (0.6-1.7		0.7 (0.5-1.2)		1.2 (0.8-2.0)	
p-value	0.4		0.8		0.2		0.3	

Household Income								
Four higher quintiles	1.0	1.0	1.0	1.0	1.0	1.0	1.0	1.0
Lowest quintile	1.6 (1.1-2.4)	1.9 (1.0-3.5)	1.5 (1.0-2.3)	1.1 (0.6-2.1)	1.5 (1.1-2.1)	0.9 (0.5-1.6)	1.4 (1.0-2.0)	1.1 (0.6-1.7)
p-value	0.007	0.05	0.05	0.5	0.04	0.5	0.05	0.9

Nationality								
Swiss	1.0	1.0	1.0	1.0	1.0	1.0	1.0	1.0
Non-Swiss	1.2 (0.8-2.0)	0.7 (0.3-1.5)	1.8 (1.1-2.8)	0.8 (0.4-1.9)	1.9 (1.1-3.3)	1.2 (0.5-2.7)	2.1 (1.2-3.2)	0.5 (0.2-1.3)
p-value	0.3	0.4	0.01	0.7	0.02	0.7	0.006	0.2

Language region								
German-speaking	1.0	1.0	1.0	1.0	1.0	1.0	1.0	1.0
French-speaking	1.0 (0.8-1.4)	1.3 (0.7-2.1)	1.7 (1.2-2.3)	1.4 (0.8-2.3)	1.4 (1.0-1.9)	1.4 (0.9-2.3)	2.1 (1.5-2.9)	1.8 (1.2-2.8)
p-value	0.8	0.4	0.001	0.2	0.03	0.2	<0.001	0.005
Italian-speaking	1.4 (0.6-2.9)	1.1 (0.4-3.3)	2.6 (1.3-5.4)	2.2 (0.8-6.1)	4.3 (2.1-8.7)	4.6 (1.5-14.3)	5.3 (2.3-12.2)	na^2^
p-value	0.5	0.8	0.006	0.1	<0.001	0.001	<0.001	

Internet use								
Rarely-never	1.0	1.0	1.0	1.0	1.0	1.0	1.0	1.0
Every day	0.7 (0.4-1.1)	0.6 (0.4-1.1)	0.8 (0.5-1.3)	0.8 (0.5-1.4)	0.6 (0.4-0.9)	0.5 (0.3-0.7)	0.7 (0.5-0.9)	0.7 (0.5-0.9)
p-value	0.1	0.08	0.4	0.4	0.009	<0.001	0.02	0.05

Reading								
Rarely-never	1.0	1.0	1.0	1.0	1.0	1.0	1.0	1.0
Every day	1.0 (0.7-1.3)	1.2 (0.8-1.9)	1.0 (0.7-1.3)	1.1 (0.8-1.7)	0.9 (0.7-1.2)	0.8 (0.5-1.3)	1.0 (0.8-1.3)	0.7 (0.5-1.1)
p-value	0.8	0.4	0.9	0.5	0.7	0.4	0.9	0.08

^1 ^adjusted for all variables displayed in the table except education

^2 ^OR = 12.7 (2.6-61.5), due to small numbers estimates become unreliable

All estimates and 95% CI are based on the pooled data for the one-year outcome conditional to previous physical activity level using the GEE model with an independent correlation structure for the within-subject association. Only observations were included with data for the preceding wave in individuals previously active in sports (n_male participants _= 951; n_female participants _= 931) or previously active (n_male participants _= 933; n_female participants _= 936)

## Discussion

The Swiss Household Panel gives the opportunity to observe changes in physical activity behavior over time and to assess potential determinants of physical activity behavior and of behavior changes. As observed in other studies [18], the data suggest an increase in inactivity levels with age for both young men and young women. In each age group, inactivity levels were lower in men than in women. The prevalence of membership in a sport club was higher among younger than among older individuals, and in each age group the proportion of members was higher in men than in women. A further finding of this longitudinal study was that, irrespective of age and gender, spontaneous changes from inactive to active and vice versa occurred between any two consecutive waves in the range of about 10% to 15%. Spontaneous changes from active to inactive were generally slightly more frequent than from inactive to active resulting in the observed overall decline in physical activity levels with age. Such spontaneous changes should be taken into account when planning physical activity intervention studies, especially regarding the calculation of sample sizes necessary to detect the expected effects.

The correlation between sport club membership and exercising in the cross-sectional analysis was not surprising in its nature [11], but in its strength (OR of 6.7 in young males and 8.1 in young females). For previously active individuals, leaving a sport club increased the risk of becoming "no sport" by a factor of 7.8 in young men and 11.9 in young women, risks comparable to the ones of permanent non-members (7.8 and 12.4, respectively). These findings can be expected because of the fact that structured sport activities are to a large extent carried out in the context of sport clubs. The lower but slightly increased adjusted risk of becoming "no sport" (women only) and becoming inactive (men only) among subjects who had joined a sport club may suggest a lag effect of previous non-membership. The most important findings of this study were the still strong effects of sport club membership on moderate physical activity with OR of 4.6 in both young men and women in the cross-sectional analysis (Table 4) and between 5.1 and 6.9 in the longitudinal analysis (Table 5). These effect sizes, derived from the multivariate models adjusting for other potential risk factors, were even bigger than the ones from the univariate analysis, indicating that it is necessary to control for possible confounding through other variables such as age or linguistic region.

The definition used for "inactive" was rather restrictive (less than one day of at least 30 minutes of moderate physical activity per week). When using a less restrictive definition (e.g. 0-1 day/week or 0-2 days/week), the direction of the associations remained the same, although their strength was generally weaker (data not presented).

The Swiss Household Panel data allow to explore longitudinal physical activity patterns and changes above the cross-sectional dimension with its inherent limitations of causal inference and potential cohort bias. Its longitudinal aspect made it possible to model the conditional impact of changes in sport club membership on physical activity status in the following wave. It cannot be excluded that the effect observed in these data may not be exclusively causal and that losing interest in physical activity may be the reason for both leaving a sport club and declines in sport and physical activity levels. While such mechanisms are likely, they do not fully explain the strength of the associations observed in this study and they do not nullify the important role of sport clubs for physical activity behavior of young men and women in Switzerland. This role includes not only the provision of the physical environment necessary for many sports, but also qualified instruction and social support.

Compared to sport club membership, all other covariates included in the multivariate model were either non-significant or had much smaller effect sizes. Age was a predictor of being "inactive", but not of becoming "inactive", suggesting a more or less continuous decline after controlling for covariates. Low household income and a foreign nationality had an influence in subgroups, but it has to be taken into consideration that the survey only included subjects able to participate in a lengthy interview in German, French or Italian. Moreover, immigrants of lower socio-economic status are expected to be under-represented. If a more restrictive definition of nationality was used, namely exclusively Swiss instead of Swiss with a second nationality versus all others, stronger associations were detected (data not shown). Sedentary behavior expressed as daily Internet use and daily reading was protective of being or becoming physically inactive rather than representing a risk. The discrepancy of these findings with other studies identifying sedentary behavior as a risk factor [18] may be due to the choice of indicators which might be correlates of higher socio-economic status or higher intellectual activity. Moreover, different types of sedentary behavior may show different associations with physical activity behavior [26]. For example, a positive correlation with physical activity has been reported for "productive" sedentary behaviors including reading/doing homework and working on a computer [23], and for reading in both young men and young women in one study [25], and in young females but not in males in another study [26].

Education was not included in the multivariate models because of its strong correlation with age. For example, compulsory school is only attended up to age 17 years, and the youngest age to start university is 18 years. However, there may also be a socio-economic influence on education, with young adults from higher socio-economic backgrounds being more likely to go to high school and university. It was beyond the scope of this article to disentangle these aspects and to look at changes in educational tracks preceding changes in physical activity behavior. Thus it was not possible to clarify the role of education as a potential confounder or as an intermediate factor on the causal pathway between age and physical activity. However, these questions may be explored in further analyses.

Strengths of the study were the large nationally representative sample, the possibility to analyze the data both cross-sectionally and longitudinally, and the number of years of follow-up. A limitation was the simple and global self-report measure used for physical activity assessment that did not allow reference to the current physical activity recommendations. Furthermore, the definition of moderate activities ("activities that get you at least a little bit out of breath") may have led to underreporting of light moderate activities. However, examples of moderate activities were provided such as brisk walking, gardening or dancing. Individuals engaging in moderate physical activity for a duration of less than 30 minutes per day could not be identified. There are no directly comparable data from the Swiss Health Survey, however the proportion engaging in at least 30 minutes of moderate physical activity on five or more days per week in the present study (8.3% in young men and 7.4% in young women) seems quite low. This was also a reason why different cut-offs were used to define active and inactive individuals, even though no reference to the physical activity recommendations was possible. It would have been ideal to include TV viewing as a sedentary activity, however data on TV viewing were only available for 1999 and could thus not be included in these analyses. An important limitation of panel data is attrition (loss-to-follow-up) which can introduce a potential bias when it occurs differentially. However, among participants followed for several waves and among those followed only for one or two waves, the level of physical activity at the initial wave was very similar indicating that there was no differential attrition with regard to physical activity. In addition, as physical activity is only a minor topic in the household panel, it is unlikely that individuals dropped out because of physical activity behavior changes. Furthermore, other factors that might cause higher drop-out rates in some population sub groups (such as socio-economic status and nationality) did not appear to be strongly correlated with physical activity behavior in our study sample.

## Conclusions

The study provides insight into the natural history of physical activity at the population level by presenting estimates of spontaneously occurring changes normally cancelled out in repeated cross-sectional surveys. In addition, it highlights the important role of sport clubs for sport activities and exercising and also for general physical activity in young people in Switzerland. More accurate measurements of physical activity and studies in other countries will allow to judge whether these findings can be generalized.

## Competing interests

The authors declare that they have no competing interests.

## Authors' contributions

DZ performed the statistical analyses and helped to interpret the results and to draft the manuscript. MW helped to write the manuscript and to interpret the results. EZ helped to perform the statistical analyses, to interpret the results and to draft the manuscript. BWM helped to interpret the results and to write the manuscript. All authors participated in the conception of the analyses, and read and approved the final manuscript.
